# Anti-Apoptotic Bfl-1 Is the Major Effector in Activation-Induced Human Mast Cell Survival

**DOI:** 10.1371/journal.pone.0039117

**Published:** 2012-06-15

**Authors:** Maria Ekoff, Katarina Lyberg, Maryla Krajewska, Monica Arvidsson, Sabina Rak, John C. Reed, Ilkka Harvima, Gunnar Nilsson

**Affiliations:** 1 Department of Medicine, Centre for Allergy Research, Karolinska Institutet, Stockholm, Sweden; 2 Sanford-Burnham Medical Research Institute, La Jolla, California, United States of America; 3 Department of Respiratory Medicine and Allergology, Sahlgrenska University Hospital, Goteborg, Sweden; 4 Department of Dermatology, Kuopio University Hospital and University of Eastern Finland, Kuopio, Finland; Otto-von-Guericke University Magdeburg, Germany

## Abstract

Mast cells are best known for their role in allergic reactions, where aggregation of FcεRI leads to the release of mast cell mediators causing allergic symptoms. The activation also induces a survival program in the cells, i.e., activation-induced mast cell survival. The aim of the present study was to investigate how the activation-induced survival is mediated. Cord blood-derived mast cells and the mast cell line LAD-2 were activated through FcεRI crosslinking, with or without addition of chemicals that inhibit the activity or expression of selected Bcl-2 family members (ABT-737; roscovitine). Cell viability was assessed using staining and flow cytometry. The expression and function of Bcl-2 family members *BFL-1* and *MCL-1* were investigated using real-time quantitative PCR and siRNA treatment. The mast cell expression of Bfl-1 was investigated in skin biopsies. FcεRI crosslinking promotes activation-induced survival of human mast cells and this is associated with an upregulation of the anti-apoptotic Bcl-2 family member Bfl-1. ABT-737 alone or in combination with roscovitine decreases viability of human mast cells although activation-induced survival is sustained, indicating a minor role for Bcl-X_L_, Bcl-2, Bcl-w and Mcl-1. Reducing *BFL-1* but not *MCL-1* levels by siRNA inhibited activation-induced mast cell survival. We also demonstrate that mast cell expression of Bfl-1 is elevated in birch-pollen-provocated skin and in lesions of atopic dermatitis and psoriasis patients. Taken together, our results highlight Bfl-1 as a major effector in activation-induced human mast cell survival.

## Introduction

Mast cells are known to be central effectors and regulators in allergic diseases. When a multivalent antigen binds to IgE occupying the high affinity receptor for IgE (FcεRI), receptor aggregation and subsequent mast cell activation occurs. This result in mast cell degranulation, changes in gene expression, and the release of inflammatory mediators causing the symptoms associated with allergic reactions [1], [2], [3]. In addition, the mast cell has the ability to survive the degranulation process, regranulate, and be activated again, which perpetuates the allergic reaction [4], [5]. One important question in mast cell biology is how mast cells survive during this degranulation – regranulation process.

It has previously been demonstrated that the aggregation of FcεRI can result in increased survival of mast cells (activation-induced survival) [6], [7], [8], [9]. Upon crosslinking of FcεRI (IgECL) murine mast cells upregulate anti-apoptotic Bcl-2 family member *A1* and *bcl-_XL_* and also to a lesser extent *bcl-2* at the mRNA level [8], [10], [11]. We have previously shown that mouse mast cells deficient in *A1* do not exhibit activation-induced survival upon IgECL [8], suggesting that *A1* is essential for this process in mouse. Similarly, the human homologue of *A1*, *BFL-1*, is upregulated in human mast cells upon IgECL [12] together with anti-apoptotic Bcl-2 family member *MCL-1* [12], [13]. These observations provide a possible explanation for IgE-mediated activation-induced mast cell survival. Furthermore, FcγRI crosslinking also induces *BFL-1* upregulation and activation-induced human mast cell survival [14], further suggesting that activation through these Fc-receptors contributes to mast cell survival.

Here we describe that Bfl-1 is a mediator of activation-induced human mast cell survival as demonstrated by siRNA experiments. We also demonstrate that activation-induced mast cell survival is sustained when the anti-apoptotic proteins Bcl-X_L_, Bcl-2, Bcl-W and Mcl-1 are targeted using inhibitors, indicating a minor role for the targeted anti-apoptotic Bcl-2 family members. Furthermore, Bfl-1 is upregulated in mast cells in various skin inflammatory models. Therefore, the observations highlight Bfl-1 as a potential target for treatment of allergic and inflammatory diseases.

## Results

### IgECL promotes activation-induced survival in cytokine deprived human mast cells

**Figure 1:**
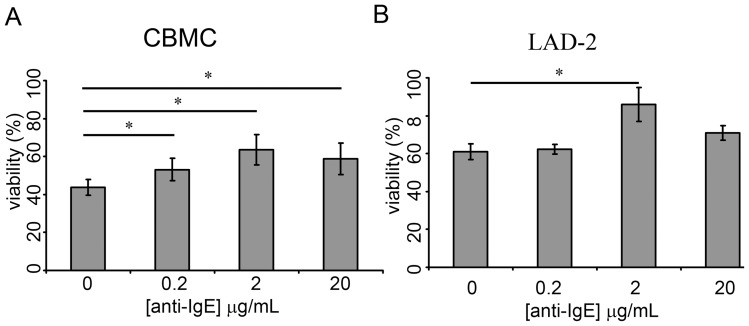
IgECL-induced survival of human mast cells. (A). CBMC upon IgECL. (B) LAD-2 cells upon IgECL. Cells were sensitized with 1 μg/mL of IgE overnight before cytokine-deprived and challenged with anti-IgE. After 24 hrs the cell viability was enumerated using trypan blue exclusion. N=3–4.

IgECL has been shown to promote survival of mast cells cultured in the absence of their required growth factors [7], [8], [9]. We therefore investigated the survival capacity of human cytokine-deprived cord blood-derived mast cells (CBMCs) and the human mast cell line LAD-2 following IgECL using a fixed concentration of human IgE (1 μg/ml) and 0.2, 2 or 20 μg/ml of anti-human IgE. The results show that IgECL resulted in prolonged survival of cytokine-deprived CBMCs for all anti-human IgE concentrations tested (Fig. 1A). Also LAD-2 cells responded with an increased survival after activation with 2 μg/ml of anti-human IgE (Fig. 1B). For further studies the concentration of 2 μg/ml of anti-human IgE was chosen since the results indicated that this concentration is superior (P=0.039 and 0.031 respectively) as compared to 0.2 and 20 μg/ml for achieving activation-induced survival of CBMCs and LAD-2 cells.

### Activation-induced mast cell survival is not dependent on Bcl-2, Bcl-X_L_, Bcl-w or Mcl-1

The family of pro-survival Bcl-2 proteins in humans consists of Bcl-2, Bcl-X_L_, Bcl-W, A1/Bfl-1, Bcl-B and Mcl-1. The activation-induced survival following IgECL is a complex process where Bcl-X_L_, Bfl-1 and Mcl-1 has been demonstrated to be induced in human mast cells, and hence have a possible role in activation-induced mast cell survival [8], [13]. To evaluate the role of Bcl-2, Bcl-X_L_, Bcl-W and Mcl-1 we used the BH3-only mimetic ABT-737 alone or in combination with roscovitine. ABT-737 is a small molecular inhibitor mimicking the binding of the BH3 domain of the pro-apoptotic protein Bad [15]. It binds with high affinity to the anti-apoptotic proteins Bcl-X_L_, Bcl-2 and Bcl-W but not Mcl-1, Bcl-B or A1/Bfl-1. The cyclin-dependent kinase (CDK) inhibitor roscovitine has been reported to down-regulate the anti-apoptotic protein Mcl-1 [16], [17], [18], [19]. The concentrations of ABT-737 and roscovitine used were chosen based on dose-response data (not shown) and with the purpose of inducing a degree of apoptosis similar to cytokine deprivation in our system.

**Figure 2:**
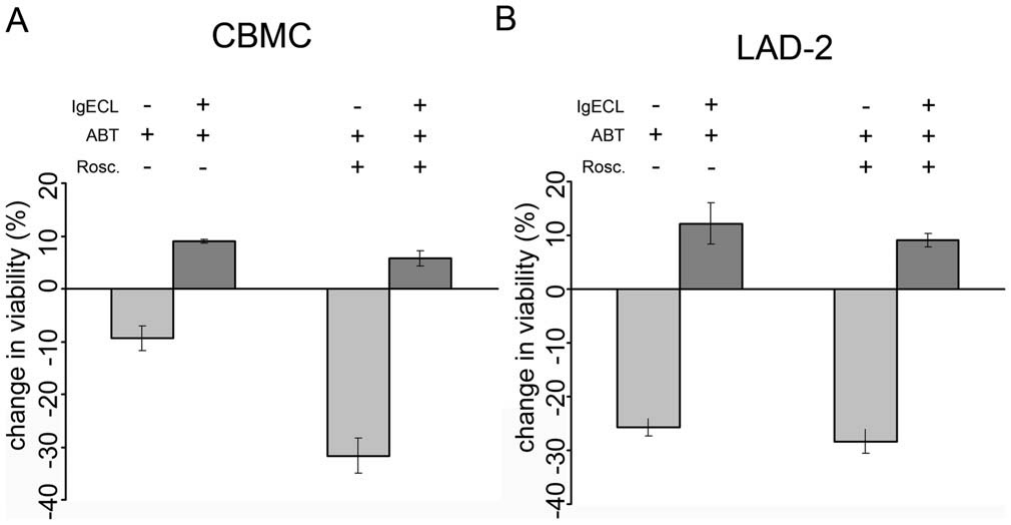
Activation-induced mast cell survival following IgECL in presence of ABT-737 and roscovitine. (A) CBMC treated with 0.5 μM ABT-737 alone or in combination with 5 μM roscovitine following IgECL. N=6–3. (B) LAD-2 treated with 0.5 μM ABT-737 alone or in combination with 5 μM roscovitine following IgECL. N=3 Viability was assessed after 24 hrs using propidium iodide plus FITC-conjugated Annexin V. Change in viability is expressed as percentage viable cells after treatment deducted from untreated cells. ABT-737 = ABT, roscovitine = rosc.

Upon treating CBMCs with 0.5 μM ABT-737, mast cell viability decreased by approximately 10% and combination of 0.5 μM ABT-737 and 5 μM roscovitine decreased this viability even further by another 20% (Fig. 2A). Decreased viability could also be observed for LAD-2 cells where the viability was decreased by approximately 25% in response to ABT-737 or ABT-737 in combination with roscovitine (Fig. 2B). The results show that IgECL resulted in prolonged survival of both ABT-737 and ABT-737/Roscovitine-treated CBMCs and LAD-2 cells (Fig. 2A–B). These results demonstrate that the ability to induce survival is sustained in an experimental setting where Bcl-2, Bcl-X_L_, Bcl-W and Mcl-1 are inhibited, and suggest that one of the remaining pro-survival Bcl-2 family members (i.e. Bfl-1 and Bcl-B) was responsible for the activation-induced survival response. Since mice lack an obvious ortholog of Bcl-B, we focused on Bfl-1, the human ortholog of A1 which was shown to be essential for murine activation-induced mast cell survival [8].

### siRNA targeting *BFL-1* inhibits activation-induced survival of human mast cells

**Figure 3:**
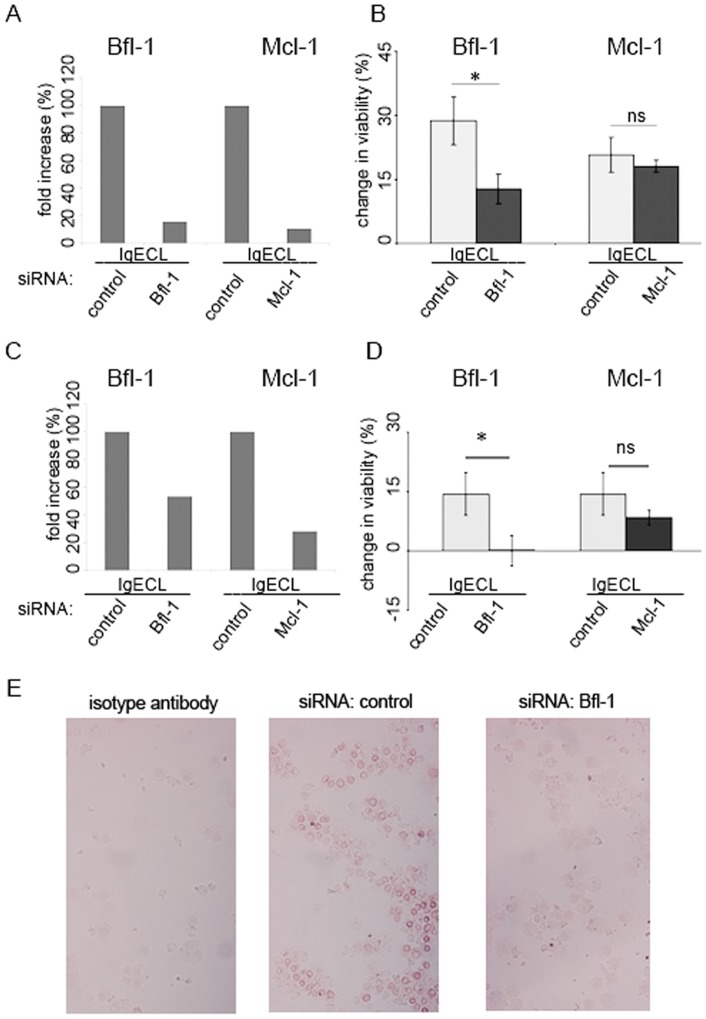
siRNA targeting *BFL-1* but not *MCL-1* diminishes activation-induced survival of human mast cells. (A) The upregulation of *BFL-1* and *MCL-1* following IgECL in CBMCs is abolished following targeted siRNA treatment as verified 30 hours post-transfection by quantative PCR. Cells were transfected with 100 nM siRNA, sensitized with 1 μg/mL of IgE and challenged with 2 μg/mL anti-IgE before expression was determined. Data correspond to one representative experiment using one donor. Similar result was obtained for another donor. (B) *BFL-1* but not *MCL-1* siRNA treated CBMCs show decreased survival upon IgECL compared to control siRNA treated cells. Cells were transfected, sensitized with 1 μg/mL of IgE and 24 hours post-transfection cytokine-deprived and challenged with 2 μg/mL anti-IgE before being enumerated 24 hours later using the vital dye trypan blue. N=6. (C) The upregulation of *BFL-1* and *MCL-1* following IgECL in LAD-2 cells is abolished following targeted siRNA treatment as described above. (D) *BFL-1* but not *MCL-1* siRNA treated LAD-2 cells show decreased survival upon IgECL compared to control siRNA treated cells. N=4. Change in viability is percentage viable cytokine-deprived cells deducted from viable cytokine-deprived cells following IgECL. (E) Bfl-1 is down-regulated in LAD-2 cells following siRNA treatment targeting *BFL-1* as compared to control siRNA in immunohistochemical stainings for Bfl-1 expression.

Having demonstrated a minor role of Bcl-X_L_, Bcl-2, Bcl-W and possibly also Mcl-1 we next examined how inhibition of *BFL-1* affects activation-induced survival of CBMC and LAD-2 cells. To inhibit gene expression, cells were electroporated using siRNA oligonucleotides targeting *BFL-1*, *MCL-1* or non-targeting siRNA. Activation-induced upregulation of both *BFL-1* and *MCL-1* at the mRNA level following IgECL were abolished by siRNA treatment, as demonstrated by quantative PCR (Fig. 3A and C). The expression of *BCL-2*, *BCL-XL*, or *C-KIT* was not affected (data not shown). In contrast, activation-induced survival was diminished by *BFL-1* targeting siRNA but not by *MCL-1* targeting siRNA (Fig. 3B and D). We could also demonstrate, by immunohistochemical staining, Bfl-1 to be down-regulated on the protein level in CBMC and LAD-2 cells by *BFL-1* siRNA treatment as compared to non-targeting siRNA (fig. 3E). Taken together our results demonstrate the critical role of Bfl-1 in prolonging the survival of cytokine-deprived human mast cells after IgECL and support our hypothesis that Bfl-1 is a major survival factor promoting activation-induced survival. Mcl-1 by comparison, seems to play a minor role in activation-induced mast cell survival, even though IgECL induces Mcl-1 expression.

### Bfl-1 is expressed in skin tissue mast cells and is increased under inflammatory conditions

**Figure 4:**
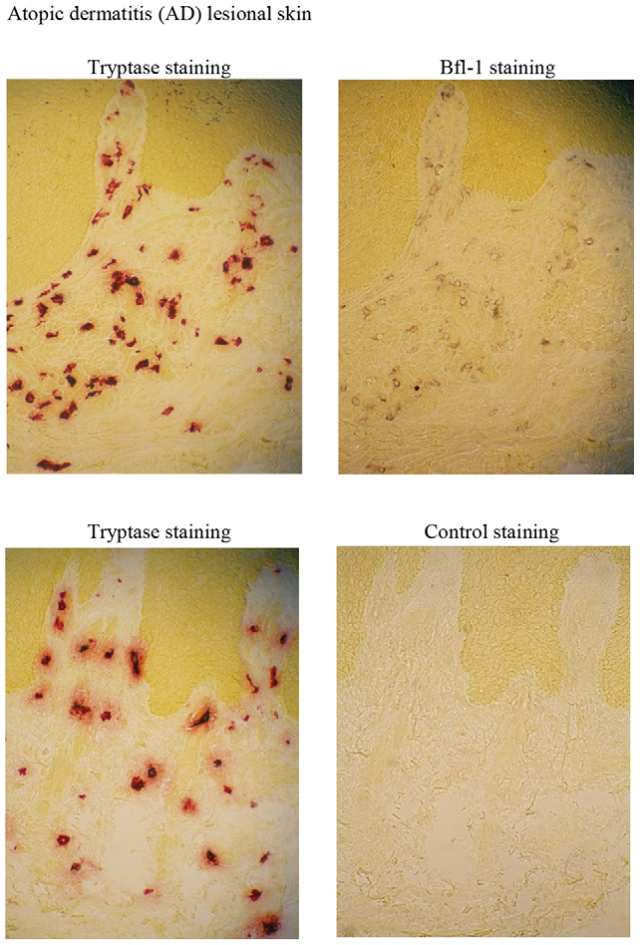
Bfl-1 is expressed in skin tissue mast cells. An enzymatical staining for tryptase followed by an immunohistochemical staining of Bfl-1 (upper panel) and was performed on atopic dermatitis (AD) lesional skin. Isotype control staining following tryptase staining (lower panel).

CBMCs as well as LAD-2 cells express tryptase and chymase (data not shown) [20], [21], [22] exhibiting a human MC_TC_ phenotype, which is the dominating phenotype in connective tissues, e.g. the skin [23]. To examine if Bfl-1 is regulated in tissue mast cells, we therefore used a doublestaining technique on skin biopsies as previously described [24]. An enzymatical staining followed by an immunohistochemical staining of the same tissue section demonstrated that Bfl-1 is expressed in skin tissue mast cells (Fig. 4).

**Figure 5:**
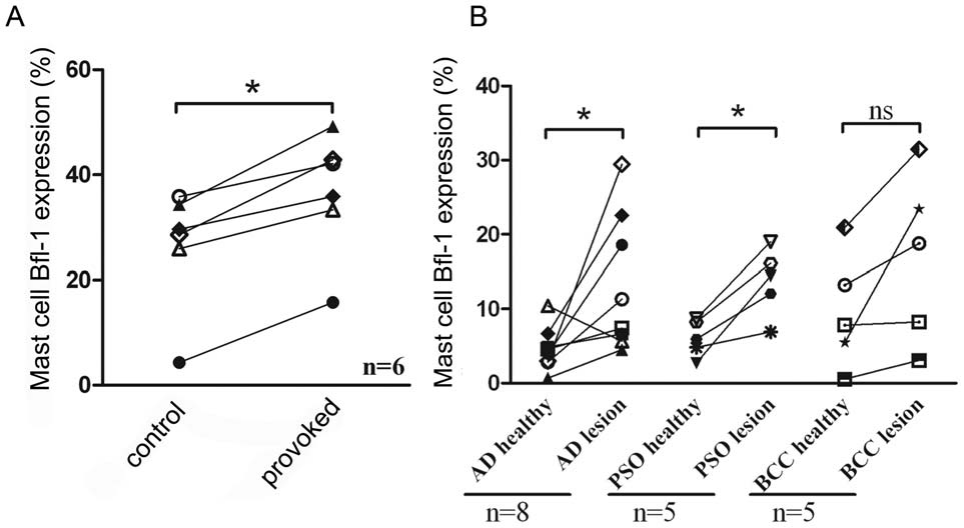
Elevated Bfl-1 expression upon allergen provocation in birch-pollen allergic patients (A) and in lesions of atopic dermatitis (AD) and psoriasis patients (PSO) (B). An enzymatical staining for tryptase followed by an immunohistochemical staining of Bfl-1 was performed. The results are presented as the percentage of mast cells expressing Bfl-1.

To examine if Bfl-1 is regulated in skin mast cells *in vivo* we performed allergen challenge on birch-pollen allergic patients. Skin biopsies were obtained 24 h following challenge with pollen allergen or diluent. A significant increase in mast cell Bfl-1 expression in the biopsies from allergen provoked skin compared with control was observed (Fig. 5a). We could not observe any significant changes in mast cell number (data not shown). Thus, IgE-receptor activated human mast cells upregulate Bfl-1 upon allergic provocation *in vivo*.

Although mast cells are perhaps best known for their involvement in allergic diseases, growing evidence also suggest that mast cells are involved in inflammatory mechanisms of chronic skin diseases. Atopic dermatitis (AD), psoriasis (PSO), and basal cell carcinoma (BCC) are chronic cutaneous inflammatory diseases where the number of mast cells is higher to varying extents in lesional compared with nonlesional skin [24], [25]. We therefore investigated if expression of the pro-survival protein Bfl-1 was increased concomitant to increased mast cell numbers in the lesional skin. A significant increase in mast cell Bfl-1 expression in the lesional skin of AD and PSO patients compared with nonlesional skin from the same patients was observed (Fig. 5b). In addition, Bfl-1 was increased in the lesional BCC skin in every case, though statistical significance was not reached (p=0.078).

## Discussion

In the present study we provide strong evidence that activation-induced human mast cell survival is dependent on the expression of Bfl-1 and that Bfl-1 is induced in skin mast cells *in vivo*.

We have previously demonstrated that, in the mouse, mRNA levels for the anti-apoptotic Bcl-2 family member *A1* are increased following IgECL and that mast cells lacking *A1* do not gain a survival advantage from IgECL [8]. Similarly, our results using quantative PCR confirm that *BFL-1* is upregulated at mRNA level following IgECL in human mast cells ([12] and this study). However, activation-induced mast cell survival following IgECL is a complex process where also other anti-apoptotic Bcl-2 family members apart from A1/Bfl-1 are regulated. In the murine system, anti-apoptotic Bcl-X_L_ is increased following IgECL [8], [26] and in human mast cells Mcl-1 [12], [13] is upregulated. Our results using the BH3 mimetic compound ABT-737 suggests a minor role of anti-apoptotic Bcl-X_L_, Bcl-2 and Bcl-W in human mast cell survival following IgECL. Furthermore, the use of ABT-737 combined with roscovitine also implicates a minor role for Mcl-1 in activation-induced survival following IgECL. In contrast, mast cell survival was impaired by *BFL-1* (but not *MCL-1*) knockdown using siRNA. Altogether, our results establish the importance of *BFL-1* for activation-induced survival of human mast cells *in vitro*.

To examine if Bfl-1 is regulated in activated mast cells *in vivo* we used human skin biopsies from birch-pollen allergic patients. We found a significant increase in mast cell Bfl-1 expression in allergen provocated skin. Although we could not detect a statistically significant change in mast cell numbers after 24 hours following allergen challenge it seems plausible that an increased mast cell Bfl-1 expression over time would lead to an increase in mast cell numbers. Thus, both our *in vivo* and *in vitro* findings suggest that activated mast cells upregulate Bfl-1 upon allergic provocation, highlighting Bfl-1 as a potential target for treatment of allergic diseases.

In addition, we also found a significant increase in mast cell Bfl-1 expression in lesional skin of AD and PSO patients compared with nonlesional skin from the same patients. FcεRI-dependent activation of mast cells is not commonly associated with these diseases, but it has been reported that in IFN-γ-rich psoriatic skin [27] mast cells express FcγRI [28]. Interestingly, we have previously shown that also FcγRI crosslinking of *in vitro*-generated human mast cells induce *BFL-1* upregulation and activation-induced mast cell survival [14]. Thus, we believe that activation-induced mast cell survival is an Fc-mediated mechanism, as we have not been able to observe this *BFL-1* upregulation and activation-induced survival for other types of mast cell stimuli, e.g. through adenosine receptors [11] or compound 48/80 [8]. Since our findings suggest that activation-induced mast cell survival is dependent on the upregulation of *BFL-1* and that there is an increase of mast cell Bfl-1 expression in lesional skin of AD and PSO patients, this could be an important explanation to increased mast cell numbers seen in AD, PSO and BCC. Still, the role of mast cells and the implications elevated Bfl-1 expression might have in mast cell survival in AD, PSO and BCC remains to be determined.

Altogether, the results reported here suggest that Bfl-1 is among the contributors to survival of mast cells, raising the possibility that agents neutralizing this Bcl-2 family member could find a role in the treatment of a wide variety of allergic and chronic inflammatory diseases.

## Materials and Methods

### Ethics statement

All patients provided written informed consent. The regional ethics review board for medical research in Gothenburg, Sweden and the etical committee of Kuopio University Hospital, Kuopio, Finland approved the studies.

### Cell cultures

Cord blood-derived mast cells (CBMCs) were derived in supplemented StemPro-34 SFM medium (Invitrogen Corp., Carlsbad, CA, USA) including 100 ng/mL recombinant human SCF (hSCF, Peprotech EC Ltd, London, UK) and 10 ng/mL human IL-6 (Peprotech EC Ltd) as previously described [12]. The human mast cell line LAD-2 (kindly provided by Drs. A. Kirshenbaum and D. Metcalfe, NIH, Bethesda, MD, USA) [22] was maintained in supplemented StemPro-34 SFM medium with 100 ng/mL hSCF.

### Mast cell activation

Cells (10^6^ cells/mL) were incubated with 1 µg/mL IgE (AG30P, Millipore, Billerica, MA, USA) overnight, washed with PBS, and activated with 0.2, 2 or 20 µg/mL anti-human IgE (Sigma, St. Louis, MO, USA) for 24 hours in medium deprived of cytokines. For inhibition studies, 0.5 µM ABT-737 (provided by Abbott laboratories) was added at the time of activation, whereas 5 µM roscovitine (Sigma) was added two hours prior activation.

### Cell survival assays

Enumeration of viable cells was performed by trypan blue exclusion or propidium iodide (PI, 2 µg/mL) and FITC-conjugated annexin V (0.3 µg/mL) analyzed by FACScan (Becton Dickinson, Franklin Lakes, NJ, USA).

### RNA preparation and real-time quantitative PCR

RNA was extracted using TriPure isolation reagent (Roche Diagnostics) according to the manufacturer's protocol. cDNA was synthesised using a First Strand cDNA Synthesis Kit (Roche Diagnosticss) and amplified using 1xSYBR Green PCR master mix (Applied Biosystems, Foster City, CA, USA) and 700 nM of primer. Relative changes in gene expression were calculated by the comparative ΔCt method in which human gyceraldehyde-3-phosphate dehydrogenase (*GAPDH*) was used for normalization. Listed 5′ to 3′, primer sequences were as follows: *BFL-1* forward, AAATGTTGCGTTCTCAGTCC; *BFL-1* reverse, AGCCTCCGTTTTGCCTTATC; *MCL-1* forward, TTACCGCGTTTCTTTTGAGG; *MCL-1* reverse CACTTCTCACTTCCGCTTCC; *GAPDH* forward, TCGGAGTCAACGGAT; *GAPDH* reverse, CTCCGACGCCTGCTT.

### siRNA-mediated inhibition of *BFL-1* and *MCL-1* gene expression

CBMC and LAD-2 cells were transfected using Human MCL1 SMARTpool siRNA Reagent, BCL2A1 SMARTpool siRNA Reagent or human ON-TARGETplus Non-targeting siRNA (Dharmacon Inc., Lafayette, CO, USA). 100 nM siRNA was introduced using a Nucleoporator (nucleofector kit VPI-1002; program X-001 (Amaxa AG, Cologne, Germany)). 24 hours post-transfection, the dead cell removal kit MACS (Miltenyi Biotec, Bergisch Gladbach, Germany) was used before anti-human IgE was added as described previously. Inhibition of *BFL-1* and *MCL-1* expression was verified by real-time quantitative PCR 30 hours post-transfection.

### Antibody generation and characterization

The anti-Bfl-1 serum (SB-50) was generated in rabbits using recombinant Bfl-1 protein. Bfl1 was produced as GST fusion protein from pGEX vectors using Escherichia coli BL21 (DE3) as the host strain. The protein purification method has been described previously [29]. New Zealand white female rabbits were injected subcutaneously with a mixture of recombinant protein (0.1 to 0.25 µg protein/immunization) and 0.5 ml Freund's complete adjuvant (dose divided over 10 injection sites), boosted three times at weekly intervals, followed by another 3 to 20 boostings at monthly intervals with recombinant protein in Freund's incomplete adjuvant, collecting blood at 1 to 3 weeks after each boosting to obtain serum.

### Detection of mast cell Bfl-1 expression

Bfl-1 expression in IgECL-activated CBMC and LAD-2 cells following siRNA treatment was investigated using an immunohistochemical staining of Bfl-1. Slides were immunohistochemically stained with an anti-Bfl-1 polyclonal rabbit antibody and the EnVision system and the 3-amino-9-etyhlcarbazole (AEC) method (Sigma) were performed according to the manufacturer's instructions (Dakocytomation, Carpinteria, CA, USA).

Bfl-1 expression in mast cells in skin inflammatory conditions was investigated in a blinded fashion using a sequential double-staining, combining an enzymatical staining for tryptase with the immunohistochemical staining of Bfl-1 [24], [30]. Tryptase containing cells were visualized using 1 mM Z-Gly-Pro-Arg-4-methoxy-2-naphthylamide (MNA) (Bachem, Bubendorf, Switzerland) and Fast Garnet GBC (Sigma) and the sections were immunohistochemically stained for Bfl-1 as described above. The mast cells were counted in a total area of 0.2–0.4 mm^2^ just beneath the epidermis.

Six birch-allergic patients with a clinical history of birch pollen-induced rhinoconjunctivitis were included in the study. The birch allergy was confirmed by positive skin prick test: 3 mm and serum-specific IgE antibodies to birch: class 2 (CAP system, Phadia, Uppsala, Sweden). Allergen challenge was performed and biopsies were collected outside birch pollen season. 50 SQ-U birch pollen allergen extracts (Aquagen® SQ *Betula verrucosa*; ALK-Abello, Hørsholm, Denmark) was injected intracutaneously in the forearm. Albumin diluent (Aquagen® SQ) was injected in the opposite forearm as a negative control. Skin biopsies were taken 24 h following challenge using a 3 mm disposable punch, under local anaesthesia with Xylocain® (Astra AB, Södertälje, Sweden). Samples were snap frozen, embedded immediately in TissueTek (Zoeterwoude, Netherlands) OCT medium and stored at −70°C. For staining, 6 μm thick sections were cut by cryostat (Leica CM1800, Nusstoch bei Heidelberg, Germany).

We also included three skin biopsy series from patients with atopic dermatitis, psoriasis, and basal cell carcinoma. The patients had not received any effective internal or UV-light treatment before biopsing. Punch biopsies (4 mm) were taken from untreated lesional skin and from healthy-looking skin at least 2 cm apart from the lesions. The biopsies were immediately embedded in OCT compound (Miles Scientific, Naperville, IL, USA) and frozen for preparation of 5-μm-thick cryosections [24], [30].

### Statistics

All data, unless otherwise stated, are presented as the mean ± SEM. The differences among different groups were determined by the Student's *t* test. *P*<0.05 was considered significant.
